# Ensemble deep learning enhanced with self-attention for predicting immunotherapeutic responses to cancers

**DOI:** 10.3389/fimmu.2022.1025330

**Published:** 2022-12-01

**Authors:** Wenyi Jin, Qian Yang, Hao Chi, Kongyuan Wei, Pengpeng Zhang, Guodong Zhao, Shi Chen, Zhijia Xia, Xiaosong Li

**Affiliations:** ^1^ Department of Orthopedics, Renmin Hospital of Wuhan University, Wuhan, China; ^2^ Clinical Molecular Medicine Testing Center, The First Affiliated Hospital of Chongqing Medical University, Chongqing, China; ^3^ Clinical Medical Collage, Southwest Medical University, Luzhou, China; ^4^ Department of General, Visceral and Transplantation Surgery, University of Heidelberg, Heidelberg, Germany; ^5^ Department of Thoracic Surgery, The First Affiliated Hospital of Nanjing Medical University, Nanjing, China; ^6^ Faculty of Hepatopancreatobiliary Surgery, The First Medical Center of Chinese People’s Liberation Army (PLA) General Hospital, Beijing, China; ^7^ Department of General, Visceral, and Transplant Surgery, Ludwig-Maximilians-University Munich, Munich, Germany

**Keywords:** deep learning, immunotherapy, cancer, PD1/PD-L1, ELISE

## Abstract

**Introduction:**

Despite the many benefits immunotherapy has brought to patients with different cancers, its clinical applications and improvements are still hindered by drug resistance. Fostering a reliable approach to identifying sufferers who are sensitive to certain immunotherapeutic agents is of great clinical relevance.

**Methods:**

We propose an ELISE (Ensemble Learning for Immunotherapeutic Response Evaluation) pipeline to generate a robust and highly accurate approach to predicting individual responses to immunotherapies. ELISE employed iterative univariable logistic regression to select genetic features of patients, using Monte Carlo Tree Search (MCTS) to tune hyperparameters. In each trial, ELISE selected multiple models for integration based on add or concatenate stacking strategies, including deep neural network, automatic feature interaction learning via self-attentive neural networks, deep factorization machine, compressed interaction network, and linear neural network, then adopted the best trial to generate a final approach. SHapley Additive exPlanations (SHAP) algorithm was applied to interpret ELISE, which was then validated in an independent test set.

**Result:**

Regarding prediction of responses to atezolizumab within esophageal adenocarcinoma (EAC) patients, ELISE demonstrated a superior accuracy (Area Under Curve [AUC] = 100.00%). AC005786.3 (Mean [|SHAP value|] = 0.0097) was distinguished as the most valuable contributor to ELISE output, followed by SNORD3D (0.0092), RN7SKP72 (0.0081), EREG (0.0069), IGHV4-80 (0.0063), and MIR4526 (0.0063). Mechanistically, immunoglobulin complex, immunoglobulin production, adaptive immune response, antigen binding and others, were downregulated in ELISE-neg EAC subtypes and resulted in unfavorable responses. More encouragingly, ELISE could be extended to accurately estimate the responsiveness of various immunotherapeutic agents against other cancers, including PD1/PD-L1 suppressor against metastatic urothelial cancer (AUC = 88.86%), and MAGE−A3 immunotherapy against metastatic melanoma (AUC = 100.00%).

**Discussion:**

This study presented deep insights into integrating ensemble deep learning with self-attention as a mechanism for predicting immunotherapy responses to human cancers, highlighting ELISE as a potential tool to generate reliable approaches to individualized treatment.

## Introduction

Avoiding immune surveillance by reconstructing the tumor microenvironment and compromising antigen presentation machinery to seize growth advantages has been widely recognized as a hallmark of human cancers (1), which makes adoptive cell transfer and therapies targeted to immune checkpoints the new therapeutic pillars within oncology (2). Many immunotherapies have received durable clinical responses, including pancreatic (3), gastric (4), bladder (5), and lung cancer (6); however, limited response rates and unclear underlying mechanisms hinder further immunotherapy development, so only subsets of cancer patients can benefit from them (7). For instance, although nivolumab renewed melanoma clinical treatment, about 39% of patients had progressed at the 5-year follow-up (8). Failure of immunotherapies to reach tumor remission is ascribed to many molecular and cellular mechanisms, such as altered tumor microenvironment (9, 10) and defects in antigen presentation machinery (11), which makes the key points of clinical success of future immunotherapeutics likely to lie in the pre-evaluation of individual responses in order to tailor strategies (9).

The emerging deep learning technologies have the potential to drive away the shadows hanging over immunotherapy and offer a glimmer of hope, since it has already powered recent disease diagnosis and prognosis prediction (12). For example, prognostication of clear cell renal cell carcinoma significantly benefits from deep learning, even in a previous study where a very simple neural network was deployed (13). The immunotherapeutic responses prediction is a classification issue that can be greatly improved with many state-of-the-art (SOTA) neural network architectures that have demonstrated their outstanding performances in computational science fields but have yet to be applied in medical areas. For example, Autoint (Automatic feature interaction learning *via* self-attentive neural networks), a deep neural network with residual connections and a multi-head self-attention, can map both numerical and categorical features into the same low-dimensional space to explicitly model the feature interactions, and has demonstrated its SOTA performance in the benchmark comparison (14). RNA-seq data are typically ultra-high-dimensional data, which are difficult to be fitted accurately by a single algorithm. Secondly, the data distribution of gene expression profiles approximates a Poisson distribution. However, considering the different sequencing platforms, the actual distribution may be a mixture of multiple distributions. Therefore, a combination of different algorithms is needed

In the present study, we proposed ELISE (Ensemble Learning for Immunotherapeutic Response Evaluation) by combining Linear Neural Network (LNN), Deep Neural Network (DNN), Deep Factorization Machine (DeepFM), Compressed Interaction Networks (CIN), and Autoint. ELISE inputted a pre-selection phase to erase irrelevant features and employed MCTS algorithm to output the best model. ELISE was validated to be a general pipeline for predicting immunotherapeutic responses to many human cancers and featured high potential for predicting any immunotherapeutic response against any tumor.

## Materials and methods

### Patients

Responses data of atezolizumab on resectable EACs were obtained *via* Gene Expression Omnibus (GEO) (GEO Access ID: GSE165252), which presented RNA expression data in the form of normalized counts. In the present study, GSE165252 were converted to TPM (Transcripts Per Million, or Transcripts Per kilobase of exon model per Million mapped reads) using R software (version 4.1.0), as normalized counts are not acceptable for any prediction models.

Responses data and RNA-seq data of PD-1/PD-L1 suppressor on metastatic urothelial cancers and MAGE−A3 on metastatic melanoma, were respectively obtained *via* GEO Access IDs GSE176307 and GSE35640.

### ELISE architecture

The feature selection phase was conducted with R software, implementing logistic regression as per our previous study (15), for selecting features that impact outcomes significantly. Features met p-value < 0.001 in their corresponding logistic regression model were retained and considered as the important features for clinical outcomes.

The remaining phases of ELISE were conducted with Python software (version 3.8). LNN and DNN are the base neural network architecture, differing in the number of hidden layers according to our previous study. LNN and DNN performed well in some cases, so both were included in ELISE (12). DeepFM combines the power of factorization machines for recommendation and deep learning for feature learning in a new neural network architecture (16). CIN aims to generate feature interactions in an explicit fashion at the vector-wise level (17). AutoInt can be applied to both numerical and categorical input features, and maps these into the same low-dimensional space. Then, a multi-head self-attentive neural network with residual connections was used to explicitly model the feature interactions in the low-dimensional space (14). All these neural networks were applied using package DeepTable in python, and MCTS used for hyperparameters tuning (github.com/DataCanvasIO/DeepTables).

For each trail in the model training, ELISE used MCTS to decide what models should be trained, and then optimized their hyperparameters based on the observation of hyperparameters optimization history. After all models were trained, they were considered as “weak learners”. ELISE stacked all predictions of “weak learners” to output final prediction.

Area Under Curve (AUC) of receiver operating characteristic curve (ROC) and calibration were employed to evaluate performance of ELISE in the test and train cohorts. These analyses were conducted in R with pROC and rms packages.

### Interpretability

SHAP provides a game theory-based approach to interpret any deep learning models’ output, connecting optimal credit allocation with local explanations using the classic Shapley values from game theory and their related extensions (18). We employed SHAP to interpret ELISE using the shap package in python.

### Dissecting molecular mechanisms

Gene set enrichment analysis (GSEA) was employed to elucidate the dysregulated biological processes, molecular functions, cellular components, and signaling pathways of ELISE subtypes. The differential expressed genes (FDR < 0.05, log_2_ Fold-Change >1) were involved in GSEA analyses. GSEA relied on Gene Ontology dataset and KEGG dataset curated in GSEA official database (19).

Estimation of stromal and immune cells in malignant tumor tissues using expression data (ESTIMATE) algorithm is a sophisticated algorithm which is designed for measuring the degree of infiltration of cancer cells and different normal cells by exploiting the unique properties of tumor cell transcriptional profiles (20), with its robustness having been validated in various cancers. The present study employed ESTIMATE algorithm which was provided by ESTIMATE package in R. This was used to quantify the global tumor microenvironment into four characterized indictors, including stromal score, immune score, ESTIMATE score, and tumor purity, representing infiltration abundance of stromal cells, immune cells, overall normal cells, and tumor cells, respectively. Since the resultant data had a skewed distribution, a grouped comparison was performed with a Wilcoxon test, and Spearman coefficients evaluated their correlation. All p-values were corrected using Benjamini–Hochberg method to avoid false positive results.

According to our previous study, we used single sample GSEA (ssGSEA) to dissect immune cell infiltration between ELISE subtypes (12).

### Statistical analysis

Raw data were collated by R software. The statistical analyses were based on R and Python software. The statistical results and interactive network data analysis were visualized with Cytoscape version 3.7.1 (Cytoscape Consortium, San Diego, California, USA). According to the previous study (15), Pearson’s and Spearman’s correlation coefficients were utilized to calculate continuous and categorical variables, respectively.

## Results

### ELISE methodology

**Figure 1:**
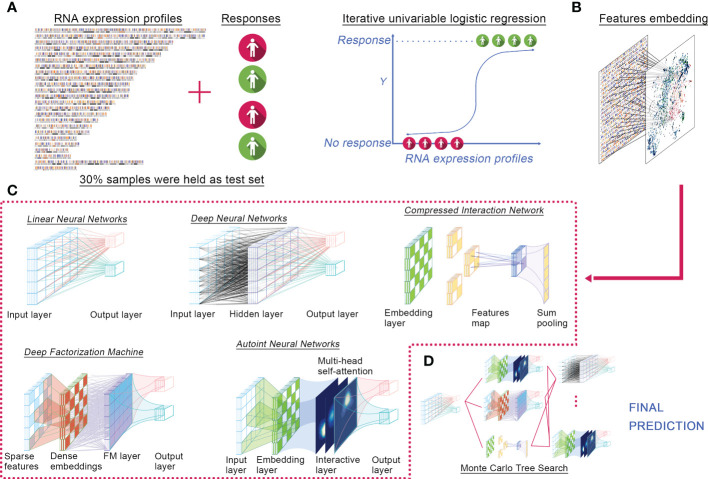
ELISE pipeline. **(A)** Inputted data. **(B)** Feature embedding. **(C)** Neural networks. **(D)** Hyperparameters tuning.

We proposed ELISE as a computerized approach for individualized prediction of immunotherapeutic response to human cancers based on their transcriptomic data (**Figure 1**). ELISE consists of four core components: Feature Selection, Feature Embedding, Deep Learning Models, and Hyperparameter Optimization modules. The collated transcriptome data and clinical immunotherapy response data were first loaded into the Feature Selection module, which employed iterative univariable logistic regression to parallelize and evaluate the impact of all input features on the outcomes, where features with *p*-values less than the pre-defined screening threshold were subsetted as input data of the next module, Feature Embedding (**Figure 1A**). Subsetted features were either directly loaded into a dense layer of the next training model, LNN, or those features were discretized or categorized to the embedding layer (**Figure 1B**). The subsequent module is Deep Learning Models (**Figure 1C**), which incorporated five of the most prevalent neural network architectures available recently, including DNN, Autoint, DeepFM, CIN, and LNN. After pre-defining the hyperparameter search space or directly adopting the default settings, the Hyperparameter Optimization module was initiated for hyperparameter optimization *via* MCTS algorithm (**Figure 1D**). In each trial, the module trained a different number of neural networks, performed individual hyperparameter tuning for each network, and subsequently stacked all networks using the concatenate or add strategy and offered the final prediction. Notably, ELISE employed a sigmoid function as the activation function, a binary cross-entropy as the loss function, an AUC as the evaluation metric, and an Adam optimizer in all trials.

ELISE was designed to process different normalized data, no matter TPM or RSEM. The potential user just needs to ensure their data in a standalone task is homogeneous, i.e., normalized *via* the same method. For evidence these hypotheses, we implemented ELISE for three different tasks. Data normalization methods among these tasks were different, but each task’s data was normalized *via* the same method to ensure their homogeneous.

### ELISE performed with outstanding accuracy in predicting atezolizumab responses to EAC

**Figure 2:**
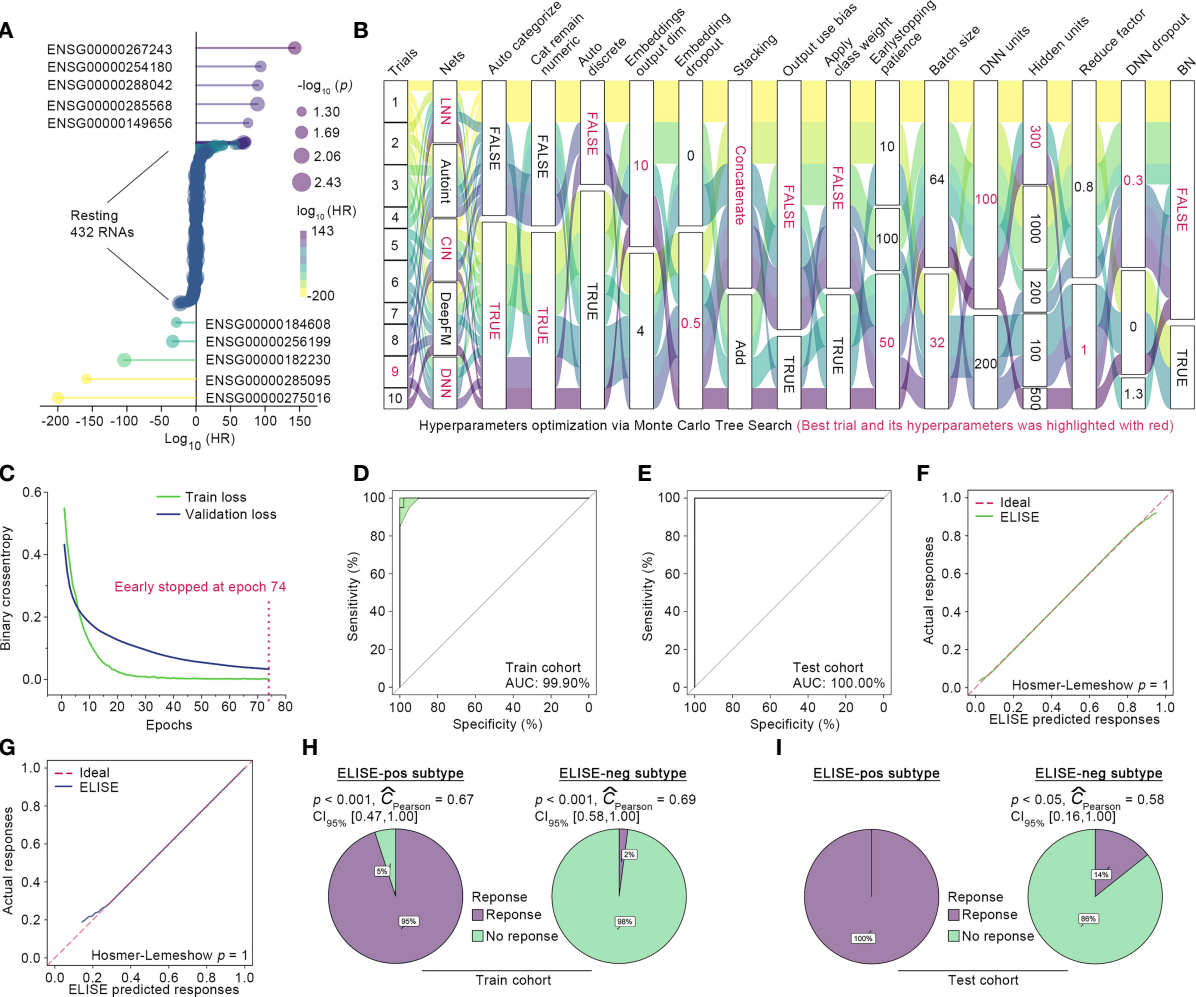
ELISE applied to EACs. **(A)** Resultant data of feature selection. **(B)** Hyperparameter optimization. **(C)** Loss curves of the best trial. **(D, E)** presented AUCs of ELISE in the train and test cohort. **(F, G)** are the calibration plot of ELISE in the train and test cohort, respectively. **(H, I)** displayed the actual outcomes distribution in the ELISE-neg and ELISE-pos subtypes.

A total of 76 EAC suffers were randomly split into initial train and test cohorts at a proportion of 8:2, and 10% of those in the initial train cohort were randomly shuffled out as the validation cohort with the remaining 90% defined as the final train cohort. Then, ELISE trained the prediction model only with the train cohort, which was validated using the validation cohort and then independently tested within the test cohort. Feature Selection module identified 442 RNAs as the most important contributors to atezolizumab responses (all *p* < 0.001, **Figure 2A**). The retained features were loaded into Deep Learning Module for launching the training process, in which the drifting features were corrected with Adversarial Validation algorithm. After ten trials, MCTS identified the ninth trial as the best trial with the smallest validation loss and relatively small train loss; the train history and the best hyperparameters are presented in **Figure 2B**. Specifically, the ninth trial was stopped early at the epoch 74 with a train loss of 0.0011 and a validation loss of 0.0329 (**Figure 2C**).

The independent test cohort was then employed for testing ELISE performance. The existing expert consensus is that a prediction model is considered to feature high discrimination when its AUC is higher than 75% (21). As expected, ELISE presented outstanding discrimination in terms of atezolizumab responses prediction, which could be evidenced by the AUC of 100.00% in the test cohort and AUC of 99.90% in the training cohort (**Figures 2D, E**). Calibration plots also demonstrated that ELISE performed a good calibration (**Figures 2F, G**), which means ELISE could correctly estimate the absolute risk (21). ELISE ultimately distinguished EAC patients into two subtypes, the ELISE-pos subtype (ELISE-identified subtype with positive response to immunotherapies) and the ELISE-neg subtype (ELISE-identified subtype with negative response to immunotherapies), in which the ELISE-pos subtypes displayed a predominant proportion of patients with immunotherapeutic response and the ELISE-neg subtype held the opposite, with most patients without an immune response (**Figures 2H, I**).

### Interpreting ELISE

Deep learning models are deemed “black boxes,” despite the good predictions made; however, it is difficult to understand the logic behind the predictions (22). The correct interpretation of these “black boxes” is of great importance, as they engender appropriate user trust and support the understanding of the process being modeled (23). However, the prevailing method to interpret deep learning or machine learning model in the medical field remains Variable Importance algorithm (12, 24), which is a biased method that fails to explain how the features affect the specific or overall predictive ability of the models (23, 24). A novel algorithm, SHAP, has been proposed to overcome these limitations (23). SHAP is a game theoretic approach to interpret the output of any deep learning model. It computes the global interpretation by calculating and combining the SHAP values for a whole dataset and measures the impact direction of each feature (23). In the present study, SHAP was used to interpret ELISE and improve the user trust of it.

**Figure 3:**
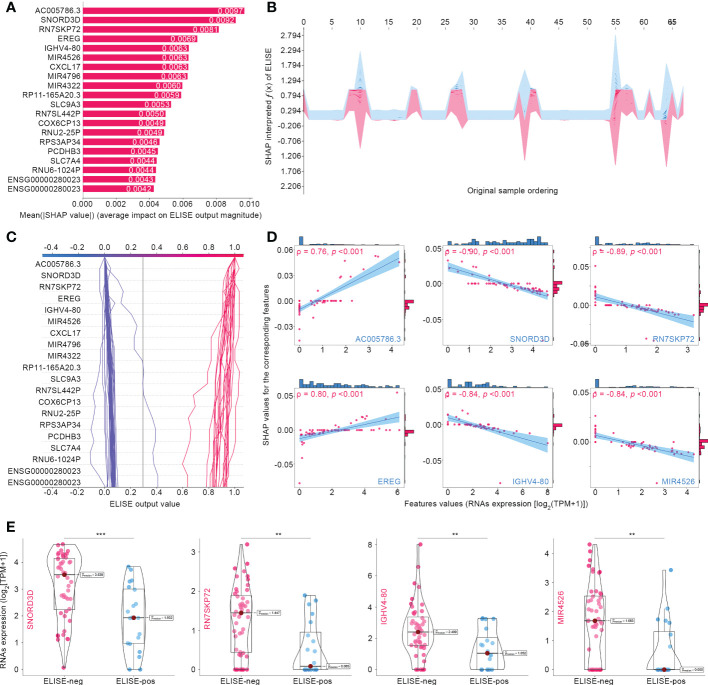
Interpreting ELISE in EACs. **(A)** SHAP summary plot ranked and presented the top 20 important features. **(B, C)** exhibited how ELISE makes the global and individual prediction. **(D)** Dependent plot indicated the affection directions of top 6 features. **(E)** Many top important features identified by SHAP presented differential expression profiles between ELISE-neg and ELISE-pos subtypes. The symbols ** represents p < 0.01, and *** represents p < 0.001.

**Figure 3A** summarizes the top 20 SHAP-identified important features, ranked according to Mean (|SHAP value|) to quantify the impact of all features on ELISE prediction (unfavorable immunotherapeutic response). *AC005786.3* (Mean [|SHAP value|] = 0.0097) was distinguished as the most valuable contributor to ELISE output, followed by *SNORD3D* (0.0092), *RN7SKP72* (0.0081), *EREG* (0.0069), *IGHV4-80* (0.0063), *MIR4526* (0.0063), etc. SHAP values includes an essential property that always sum up the difference between the players-present and players-absent game outcomes. For ELISE, a deep learning model, SHAP values of all the input features will always sum up to the difference between baseline (expected) and real-time ELISE outcomes for the prediction being explained (25). Thus, the SHAP algorithm interpreted how ELISE summed up each features’ contribution and made the final predication accordingly. The stacked force plot presented in **Figure 3B** displayed features contributing to pushing the ELISE individual prediction from the base value (the mean ELISE prediction over the train set) to the final prediction (features pushing the prediction higher are marked in red and those pushing the prediction lower are in blue). The decision plot in **Figure 3C** further highlights the contributions of the top 20 features’ observed values to ELISE outputs and how they push the model prediction in each sample. The impacts of the top six features on the output of ELISE were further quantified with dependent plots (**Figure 3D**); *AC005786.3* and *EREG* demonstrated negative contributions to ELISE, predicting poor responses to immunotherapies, with Spearman’s ρ to their SHAP values of 0.73 and 0.80, respectively. On the contrary, *SNORD3D*, *RN7SKP72*, *IGHV4-80*, and *MIR4526* raised risk to unfavorable responses, which were evidenced by their Spearman’s ρ of -0.90, -0.89, -0.84, and -0.84, respectively. *SNORD3D*, *RN7SKP72*, *IGHV4-80*, and *MIR4526* held much higher expression profiles within ELISE-neg subtypes than other ELISE-pos counterparts (**Figure 3E**), which was in the line with resultant data of dependent plots and reaffirmed that they served as risk factors to poor responses to immunotherapies given to EAC patients.

### Molecular mechanisms leading to poor responses to atezolizumab in ELISE-neg EAC subtype

**Figure 4:**
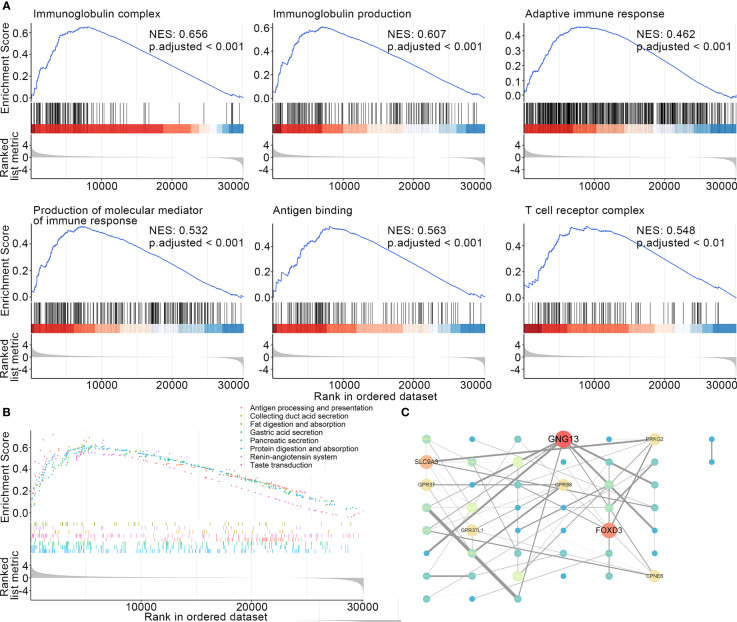
Dissecting underlying mechanisms leading to different outcomes. **(A)** Resultant data of GSEA (BP, CC, MF). **(B)** GSEA results (signaling pathways). **(C)** Protein-protein interaction network.

It is of great clinical relevance to provide deep insight and elucidate the molecular mechanisms underlying the failure to immunotherapies in the ELISE-neg EAC subtype. GSEA was employed to offer an atlas of dysregulated biological processes, molecular functions, cellular components, and signaling pathways of ELISE subtypes. As resultant data shown in **Figure 4A**, certain critical biological processes, molecular functions, and cellular components involved in immunosurveillance and the cytotoxic effect mediated by immune cells towards human EAC, including immunoglobulin complex (NES: 0.656, adjusted < 0.001), immunoglobulin production (NES: 0.607, adjusted < 0.001), production of molecular mediator of immune response (NES: 0.532, adjusted < 0.001), adaptive immune response (NES: 0.462, adjusted < 0.001), antigen binding (NES: 0.563, adjusted < 0.001), and T cell receptor complex (NES: 0.548, adjusted < 0.001), were upregulated in ELISE-pos EAC and downregulated in ELISE-neg EAC subtypes. Further GSEA to analyze dysregulated signal pathways also revealed that key immune pathways were enriched in ELISE-pos EAC subtypes, which were downregulated in ELISE-neg subtypes, such as antigen processing and presentation (**Figure 4B**), and the proteins encoded by key RNAs involved in the ELISE model had significant interactions (**Figure 4C**). These positive findings strongly indicate that critical molecules synergistically mediate the downregulation of immune signaling and result in failure of atezolizumab treatment on EACs.

**Figure 5:**
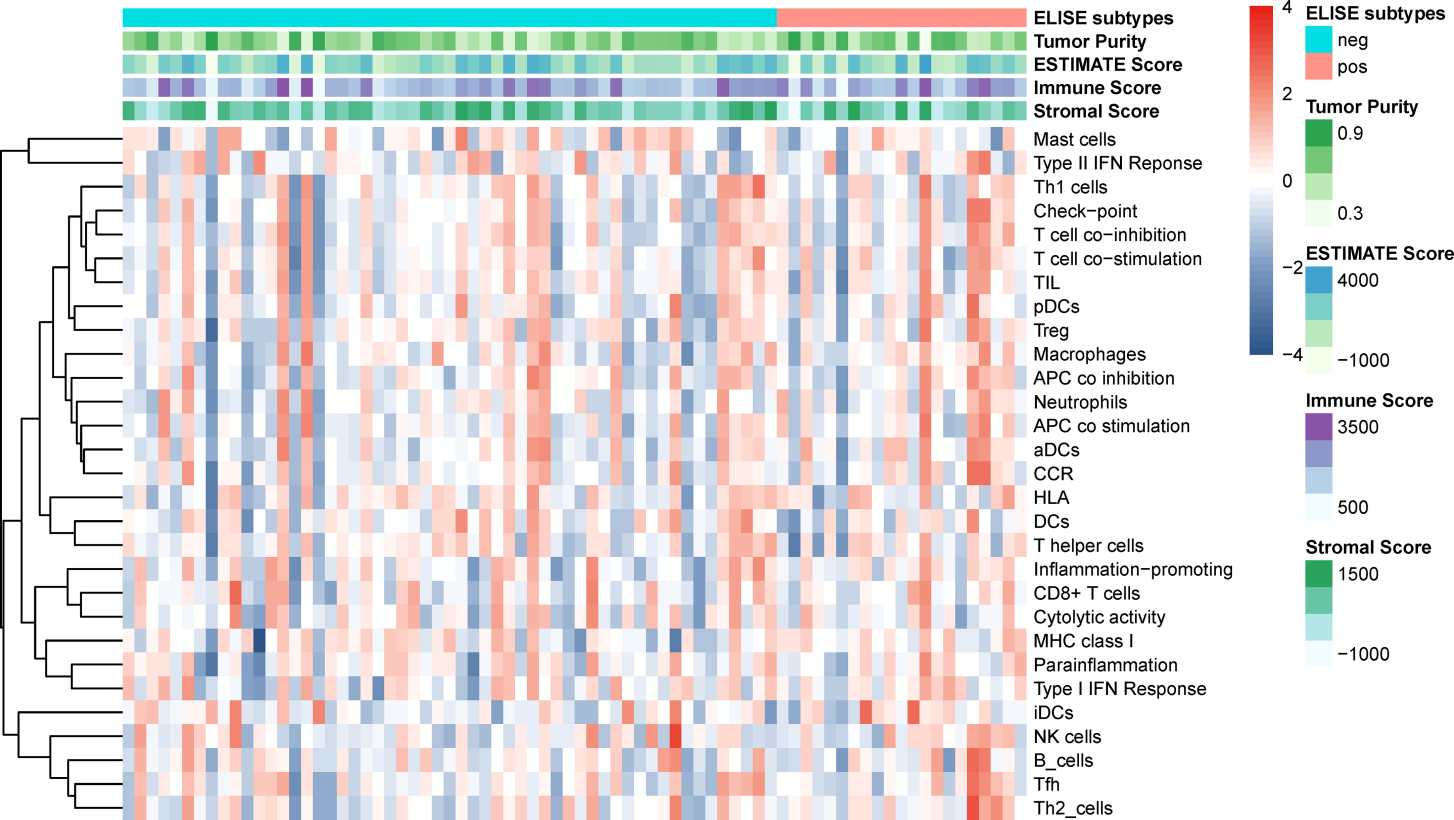
Tumor microenvironments and immune cell infiltration.

Since the immune microenvironment in EACs plays a vital role in their tumorigenesis, malignant progression, and remote migration, we further investigated the tumor microenvironment of ELISE-subtyped EACs. However, as displayed in **Figure 5**, tumor purity, immune microenvironment, 29 types of immune cellular component infiltrations, and stromal cells infiltration, did not show any significant differences between ELISE-pos and ELISE-neg subtypes. These results indicate that atezolizumab may affect the immune microenvironments less, but still affects immune cell functions and their downstream pathways in EACs.

### ELISE is a general pipeline to predict immunotherapeutic responses to human multi-cancers

To evidence the general applicability and robustness of ELISE in the prediction of pan-cancer responses to immunotherapies, it was tested in two different human cancers, metastatic urothelial cancer (UC) and metastatic melanoma.

**Figure 6:**
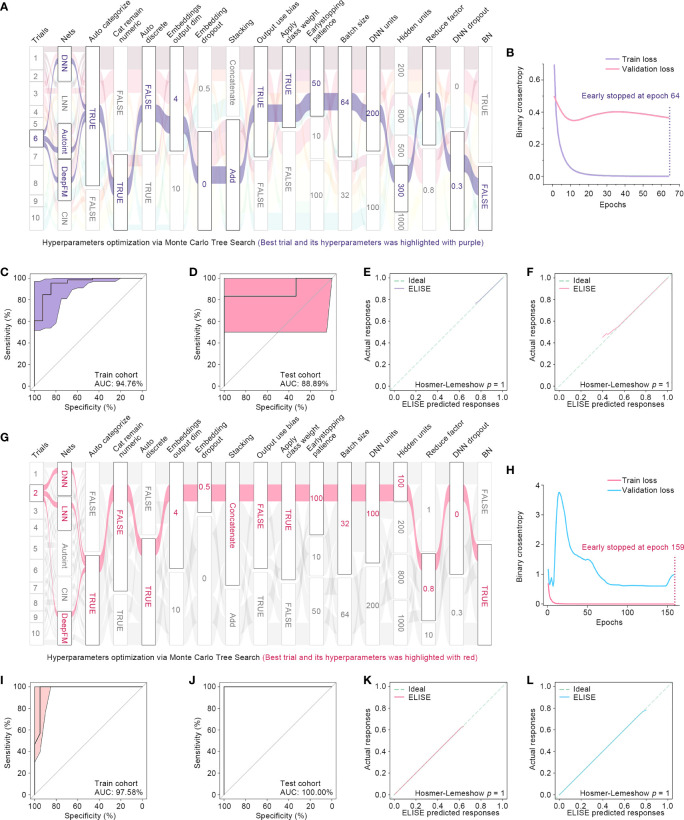
ELISE applied in UCs and melanoma. **(A)** Hyperparameter optimization in UCs. **(B)** Loss function curves in UCs. **(C, D)** AUCs of ELISE applied in UCs in the train and test cohort. **(E, F)** are the calibration plots of ELISE in the train and test cohort, respectively. **(G)** Hyperparameter optimization in MELANOMAs. **(H)** Loss function curves in MELANOMAs. **(I, J)** AMELANOMAs of ELISE applied in MELANOMAs in the train and test cohort. **(K, L)** are the calibration plots of ELISE in the train and test cohort, respectively.

For prediction of responses to PD1/PD-L1 suppressor against metastatic UCs, ELISE included 89 subjects’ RNA expression profile data, in which 70% were assigned to a train cohort, 10% to a validation cohort, and the remaining 20% to an independent test cohort. During the feature selection phase, ELISE distinguished 624 RNAs as the most critical factors that caused high responses to immunotherapies of metastatic UCs (**Table S1**) that were then fed into the model training phase. ELISE employed 10 trials to select and ensemble a final prediction model and chose Trial 6 as the best trial (**Figure 6A**). The best hyperparameters included: models ensembled (DNN, Autoint, and DeepFM), parameters activated (Auto Categorization, Cat Remaining Numeric, Output Use Bias, Class Weight), parameters disabled (Auto Discrete, Batch Normalization), Stacking by Add, Embedding Output Dim of 4, Embedding Dropout of 0, Early Stopping Patience of 50, Batch Size of 64, DNN Units of 200, Hidden Units of 300, Reduce Factor of 1, DNN Dropout of 0.3, and Space Vectors of [21, 1, 1, 0, 0, 0, 0, 1, 1, 1, 1, 1, 2, 0, 1, 0]. Loss curves of Trial 6 are presented in **Figure 6B**, and early stopping was activated at Epoch 64. Finally, ELISE performed well in pre-evaluating responses to PD1/PD-L1 suppressor against metastatic UCs, which could be evidenced by high AUCs in the train cohort (**Figure 6C**) and the test cohort (**Figure 6D**). ELISE prediction, also as expected, was highly in line with the actual responses to PD1/PD-L1 suppressor upon metastatic UCs in the train cohort (**Figure 6E**) and the test cohort (**Figure 6F**).

When applied to foresee MAGE−A3 responses to metastatic melanoma, ELISE also performed outstandingly. ELISE trained a prediction model with RNA expression data of 56 melanoma suffers, in which 713 RNAs were identified as the most significant impactors for raising unfavorable responses to MAGE−A3 treatment (**Table S2**). After all trials were completed, Trial 2 was triumphed as the best trial (**Figure 6G**), offering the best hyperparameters of models ensembled (DNN, LNN, and DeepFM), parameters activated (Auto Categorization, Auto Discrete, Class Weight, Batch Normalization), parameters disabled (Cat Remaining Numeric, Output Use Bias), Stacking by Concatenation, Embedding Output Dim of 4, Embedding Dropout of 0.5, Early Stopping Patience of 100, Batch Size of 32, DNN Units of 100, Hidden Units of 100, Reduce Factor of 0.8, DNN Dropout of 0, and Space Vectors of [25, 1, 0, 1, 0, 1, 1, 0, 1, 2, 0, 0, 0, 1, 0, 1]. ELISE reached high AUCs in the train and test cohorts of 97.58% and 100%, respectively, which demonstrated that ELISE presented high discrimination in predicting MAGE−A3 responses against metastatic melanoma.

**Figure 7:**
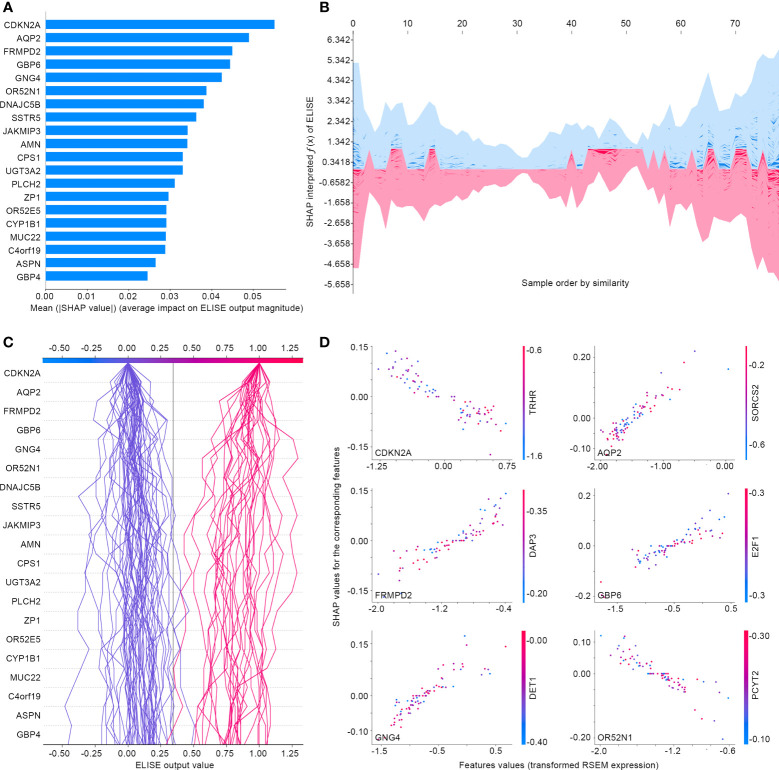
Interpreting ELISE in UCs. **(A)** SHAP summary plot ranked and presented the top 20 important features. **(B, C)** exhibited how ELISE makes the global and individual prediction. **(D)** Dependent plot indicated the affection directions of top 6 features.

Subsequently, ELISE was tailored to predict responses to PD1/PD-L1 suppressor against metastatic UCs, picked for interpretation owing to the large sample size of the metastatic UCs cohort, to demonstrate the general interpretability of ELISE in the human pan-cancers. As demonstrated in **Figure 7A**, SHAP algorithm ranked all inputted features according to their mean |SHAP values| to discover important features that made the decisive contributions to unfavorable immunotherapeutic responses. *CDKN2A* was outstanding as the most pivotal contributor with the highest mean |SHAP values| of 0.0551, followed by *AQP2*, *FRMPD2*, *GBP6*, *GNG4*, *OR52N1* etc. Furthermore, SHAP algorithm stacked contributions of all participants to directly visualize how ELISE made the individualized prediction according to the original inputted features values, shown in **Figures 7B, C**. As learned from **Figure 7D**, *AQP2*, *FRMPD2*, *GBP6*, and *GNG4* served as catalysts to increase resistance to PD1/PD-L1 suppressor within metastatic UCs. Conversely, *CDKN2A* and *OR52N1* declined the PD1/PD-L1 resistance, which demonstrates that suffers with metastatic UCs will be more sensitive to PD1/PD-L1 suppressor with the increased expression of *CDKN2A* and *OR52N1*.

## Discussion

The present study conducted based on real-world patient data represents, to the best of our knowledge, the first attempt to develop a general pipeline for predicting responses of various immunotherapies against human pan-cancers. The contribution of our findings to the related scientific fields is not only the proposed ELISE pipeline that has already been attested for its generalization and robustness, but also offers an interpretable tool that could highly foster user trust and has the prevailing advantages for clinical application. With the assistance of the present ELISE, oncologists and clinicians will be able to pre-evaluate individual responses to specific immune treatment more rapidly and decide on tailored therapeutic approaches with a high confidence level provided by ELISE.

As a state-of-the-art bioinformatic tool, deep learning has achieved an overwhelming advantage in disease diagnosis and treatment response prediction (12, 15, 26). Traditionally, diagnosing cancers relies highly on histopathology or cytopathology, which mainly involves assessment under microscopy to detect aberrant cells within a clinical sample, evaluate biomarkers of certain cancers and determine cancers’ subtype, stage, and grade (27, 28). However, the high-throughput feasibility and reliability of such approaches has been compromised by their nature of labor-intensive and human subjectivity (29). Benefiting from deep learning, clinicians are now able to automatically or semi-automatically stage many malignant tumors, including prostate (30), colon (31), and skin cancer (32), with comparable accuracy to pathologists. Notably, deep learning plays a critical role in cancer treatment decisions that cannot be ignored, as one of the promises of precision oncology is individualizing treatment to achieve tumor remission and prolong the overall survival of patients (29, 33, 34). A large-scale study investigated over 650 drug sensitivity data on thousands of cell lines and raised a deep learning tool called “DrugCell”, which is designed as an interpretable model to predict response to therapies and is successfully validated with *in vitro* and *in vivo* data (35). More encouragingly, deep learning techniques have raised many opportunities to discover and identify drugs sensitive to human cancers, such as cimetidine sensitive to lung adenocarcinoma (36), emetine to atypical meningiomas (37), and vinorelbine to *TTN*-mutated tumors (38). These enlightening shreds of evidence prove that deep learning could be greatly beneficial in predicting immunotherapeutic responses.

With these exciting techniques, we propose ELISE, one of the present study’s most important findings, for offering highly accurate pre-evaluation of immunotherapeutic responses. ELISE powers many immune treatments for human cancers, and theoretically could be employed for predicting any immunotherapeutic response against any tumor. Taking EACs as an example, ELISE demonstrated high discrimination when employed to predict atezolizumab responses (AUC = 100.00% in the test cohort). When applied to predict other immunotherapies on different tumors, including PD1/PD-L1 suppressor against metastatic UCs and MAGE−A3 responses to metastatic melanoma, ELISE also performed outstandingly, which could be evidenced by our findings in **Figure 6**. Compared to other studies, ELISE exhibited its overwhelming advantages in terms of therapeutic outcomes prediction. For predicting atezolizumab responses of EAC patients, ELISE reached AUC value of 100.00%, yet previous study only achieved AUC value less than 80.00% (39). These positive results are attributed to the design of the ELISE pipeline. ELISE employed feature selection and feature embedding modules to pre-erase “noise” i.e., features with less or no influences upon outcomes, ensembled many state-of-the-art deep learning networks architecture including LNN, DNN, Autoint, DeepFM, and CIN, and implemented a state-of-the-art hyperparameters optimization algorithm, MCTS, for tuning hyperparameters and stacking networks to generate the best model. Moreover, ELISE does not require specific data normalization processes if batch effects are pre-removed; in fact, it can process any RNA expression data, regardless of TPM data (**Figure 3**) or RSEM data (**Figure 6**).

Autoint is a deep neural network with residual connections and multi-head self-attention; it works with the same low-dimensional space, which is mapped from both numerical and categorical features, to explicitly model the feature interactions (14). With the assistance of multi-head self-attentive neural networks, Autoint can further refine interactions of high-order features and satisfactorily fit large-scale RNA expression data in an end-to-end fashion (14). DeepFM, which was designed as an end-to-end wide & deep learning framework for CTR prediction, offers a novel, state-of-the-art neural network architecture that integrates factorization machines and deep learning for recommendation and feature learning (16). CIN aims to generate feature interactions in an explicit fashion at the vector-wise level (17). Besides, DNN and LNN have widely been employed for modeling medical data and reached remarkable performances in many cases (12, 38). Furthermore, for hyperparameters tuning, MCTS is a notoriously advanced algorithm that has led to remarkable successes of many landmark artificial intelligences, including AlphaGo. In the ELISE pipeline, all these state-of-the-art network architectures and hyperparameters optimization method were included, which endowed ELISE with outstanding performance when predicting immunotherapeutic responses to cancers.

ELISE allows model interpretation *via* SHAP algorithm to transparentize the decision process of the “black box” model and increase clinician trust. Taking metastatic UCs as an example, SHAP algorithm elucidated each inputted features’ contribution to ELISE output, determined their affecting direction, and offered the global and individual interpretation for ELISE decision processes. *CDKN2A*, in the present study, was distinguished as the most important contributor with a negative correlation to unfavorable responses to PD1/PD-L1 suppressor, consistent with previous publications. *CDKN2A* encodes p16, an endogenous inhibitor of the cyclin-dependent kinases CDK4 and CDK6, which restrict the G1/S phase transition and induce cell senescence (40). A large-scale clinical study attested that *CDKN2A* is identified as a significant transcriptional correlate of response, highlighting the association of non-immune pathways to the outcome of checkpoint blockade (41). These data emphasize the high consistency that ELISE provides to prior experiences of routine clinical practices and lab works.

The present study is limited due to the inherent disadvantages of retrospective cohort studies, and ELISE warrants further validation and improvement in large and well-designed prospective clinical trials. Moreover, the potential of ELISE is limited by the samples size, despite we searched the related dataset as much as possible. A well-designed study will be conducted if more samples are obtained in the future. Besides, we could not access survival difference between different ELISE group due to their survival data was not available. The survival analyses will be preformed as planed if more survival data is available.

## Data availability statement

The original contributions presented in the study are included in the article/**Supplementary Material**. Further inquiries can be directed to the corresponding authors.

## Author contributions

Participated in study conception design: WJ, ZX. Data analysis: WJ, QY, HC, PZ, KW and GZ. Wrote or contributed to the writing of the manuscript: WJ, QY, HC, SC, ZX and XL. Obtained the funding: XL. All authors contributed to the article and approved the submitted version.

## Funding

This study was funded by the National Natural Science Foundation of China (grant number 81871653), the Natural Science Foundation of Chongqing (cstc2020jcyj-msxmX0159), Chongqing Science and Health Joint Medical High-end Talent Project (2022GDRC012), Science and Technology Research Program of Chongqing Municipal Education Commission (KJZD-K202100402, KJQN201900449) and CQMU Program for Youth Innovation in Future Medicine (W0073).

## Conflict of interest

The authors declare that the research was conducted in the absence of any commercial or financial relationships that could be construed as a potential conflict of interest.

## Publisher’s note

All claims expressed in this article are solely those of the authors and do not necessarily represent those of their affiliated organizations, or those of the publisher, the editors and the reviewers. Any product that may be evaluated in this article, or claim that may be made by its manufacturer, is not guaranteed or endorsed by the publisher.
